# Massive Pulmonary Embolism in the Postpartum Period as the First Manifestation of Antiphospholipid Syndrome: A Case Report

**DOI:** 10.7759/cureus.97497

**Published:** 2025-11-22

**Authors:** Emilio J Davila Alvarez, Mauricio A Manzanarez Balladares, Maria Sonia Rodriguez

**Affiliations:** 1 Obstetrics and Gynecology, Hospital Militar Escuela Dr. Alejandro Dávila Bolaños, Managua, NIC; 2 Internal Medicine and Critical Care, Hospital Militar Escuela Dr. Alejandro Dávila Bolaños, Managua, NIC; 3 Maternal-Fetal Medicine, Hospital Militar Escuela Dr. Alejandro Dávila Bolaños, Managua, NIC

**Keywords:** alteplase, antiphospholipid syndrome, catheter-directed therapy, critical care, interventional cardiology, maternal health, postpartum, pregnancy, pulmonary embolism, thrombectomy

## Abstract

Pulmonary embolism (PE) in the postpartum period is a rare but potentially fatal complication. Physiologic hypercoagulability combined with obstetric comorbidities increases thrombotic risk, while underlying autoimmune causes such as antiphospholipid syndrome (APS) may further amplify this risk. We report the case of a 28-year-old woman, four weeks post-cesarean delivery for preeclampsia and intrauterine growth restriction, who presented with acute dyspnea, chest pain, and hypoxemia. Initial non-contrast chest CT suggested pneumonia, and she was treated with intravenous antibiotics; however, persistent hypoxemia prompted further evaluation. Computed tomography pulmonary angiography revealed extensive bilateral pulmonary emboli consistent with massive PE. Given the high postpartum bleeding risk, catheter-directed mechanical thrombectomy with low-dose local alteplase infusion was performed, resulting in rapid hemodynamic improvement without hemorrhagic complications. Immunologic evaluation confirmed APS based on persistent anticardiolipin and anti-β2 glycoprotein I antibody positivity. Long-term anticoagulation and hydroxychloroquine were initiated, and the patient remained clinically stable on follow-up. She subsequently achieved a new pregnancy under low-molecular-weight heparin prophylaxis and delivered without thromboembolic complications. This case highlights the importance of early consideration of thromboembolism in postpartum patients with respiratory compromise, even when initial findings suggest pneumonia. Catheter-directed therapy may be a safe and effective alternative to systemic thrombolysis in postpartum women at high bleeding risk. Early recognition, multidisciplinary management, and targeted thromboprophylaxis are essential to optimize outcomes in patients with APS-associated PE.

## Introduction

Pulmonary embolism (PE) is one of the leading direct causes of maternal morbidity and mortality worldwide [1]. During pregnancy and the postpartum period, physiologic changes such as increased coagulation factors, venous stasis, and endothelial injury fulfill Virchow’s triad, significantly increasing thromboembolic risk. The risk of PE is estimated to be 20- to 60-fold higher compared with non-pregnant women of reproductive age [2], particularly during the first six postpartum weeks.

Antiphospholipid syndrome (APS) is an autoimmune thrombophilia characterized by venous or arterial thrombosis and/or obstetric morbidity in the presence of persistent antiphospholipid antibodies-anticardiolipin (aCL), lupus anticoagulant (LA), or anti-β2 glycoprotein I (anti-β2GPI) [3]. In reproductive-aged women, APS often manifests as recurrent miscarriage, preeclampsia, intrauterine growth restriction (IUGR), or thromboembolic events [4].

Although the relationship between pregnancy and thromboembolism is well recognized, massive postpartum PE as the first clinical manifestation of APS is exceedingly rare. Prompt recognition and early multidisciplinary management are essential to improve maternal outcomes. Catheter-directed interventions have emerged as effective and safer alternatives to systemic thrombolysis, particularly in postpartum patients at high hemorrhagic risk [5,6]. Timely recognition of PE in postpartum patients also requires adherence to updated diagnostic pathways [7].

We present a case of massive postpartum PE as the initial manifestation of APS, highlighting diagnostic challenges, therapeutic decision-making, and the importance of multidisciplinary care.

## Case presentation

A 28-year-old woman, gravida 2 para 1, with a history of one prior spontaneous miscarriage, developed preeclampsia, gestational diabetes, and intrauterine growth restriction (IUGR) during her second pregnancy. She underwent cesarean delivery due to severe preeclampsia and IUGR. The newborn weighed 1,580 g with Apgar scores of 8 and 9; despite initial stability, the neonate died shortly after birth, consistent with severe placental dysfunction.

Initial presentation

Several weeks postpartum, the patient reported having experienced low-grade fever, rhinorrhea, and a moist cough without sputum, for which she did not seek medical attention. Days later, she developed sudden dyspnea, pleuritic chest pain, and palpitations.

**Figure 1:**
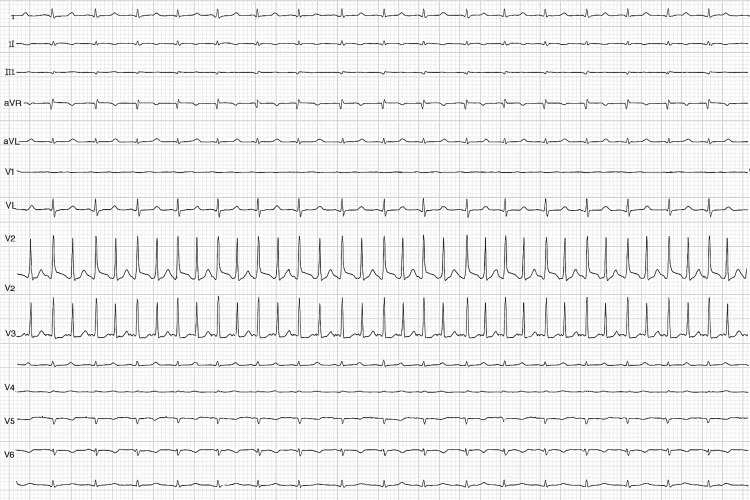
Electrocardiogram on admission Electrocardiogram on admission showing sinus rhythm, normal electrical axis (11°), PR interval of 0.12 s, QT interval of 0.32 s, and no ST-segment deviations.

In the emergency department, she was tachycardic (128 bpm), tachypneic (34/min), normotensive, and hypoxemic (SpO₂ 86% on room air). Supplemental oxygen at low flow produced only partial improvement. Lung auscultation revealed bilateral crackles, more prominent at the left base. Electrocardiography showed sinus rhythm with normal axis and intervals (Figure 1).

Laboratory tests showed leukocytosis (19,000/µL), thrombocytosis (611,000/µL), and elevated CRP (2.05 mg/dL). Arterial blood gas on supplemental oxygen demonstrated moderate-severe hypoxemia (PaO₂/FiO₂ < 200). Serum lactate was mildly elevated (2.4 mmol/L).

**Figure 2:**
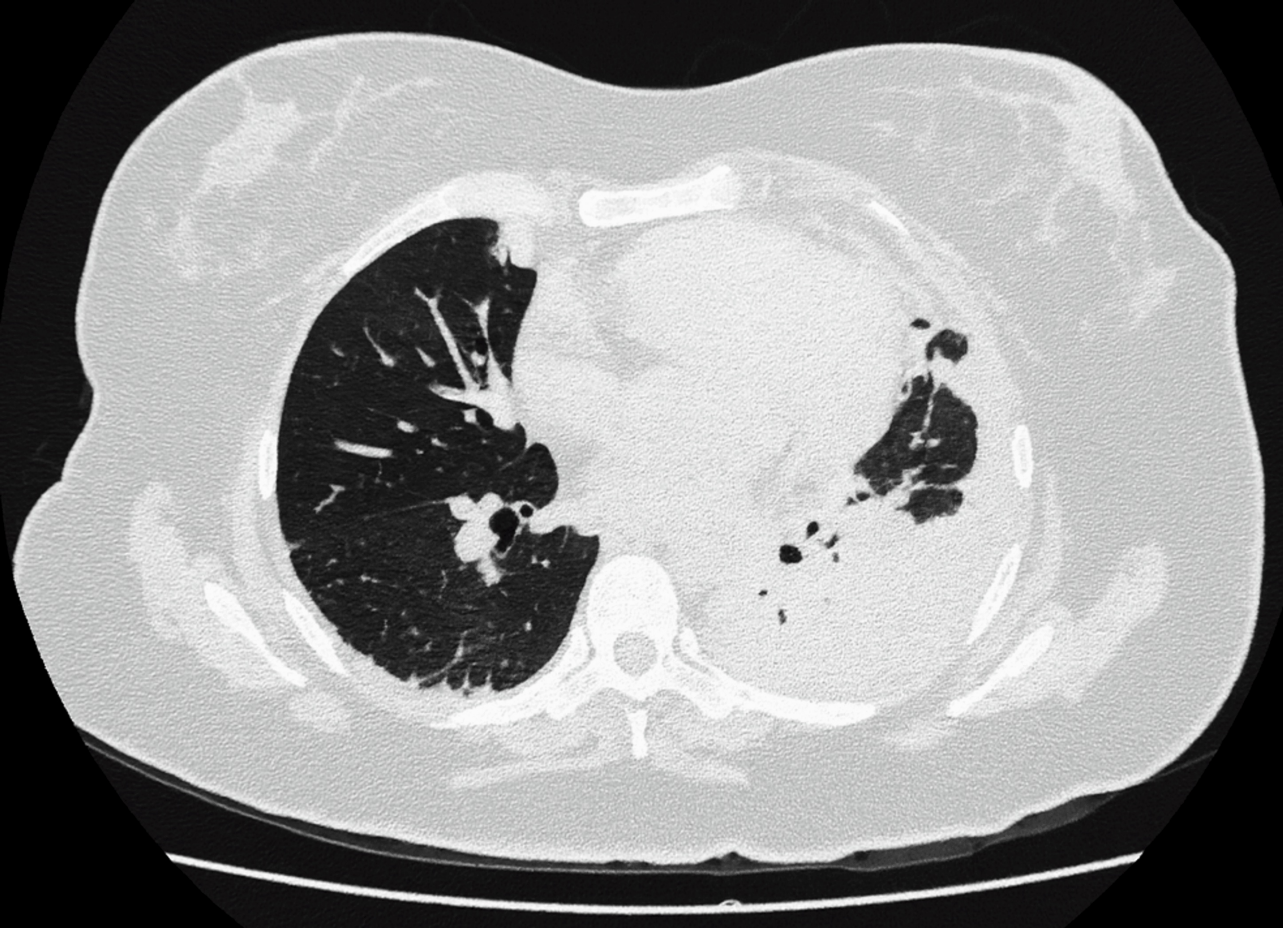
Non-contrast chest computed tomography (CT) scan Non-contrast chest computed tomography (CT) scan showing a left pleural effusion with loculated features and basal consolidation in the left lower lobe, along with ill-defined areas of consolidation in the right lower lobe.

A non-contrast chest CT, performed due to high suspicion of pneumonia, showed a loculated left pleural effusion with adjacent consolidation and ill-defined right lower-lobe opacities (Figure 2). Intravenous levofloxacin 750 mg every 24 hours was initiated, and she was admitted to the ICU due to persistent respiratory distress.

Continued evaluation

**Figure 3:**
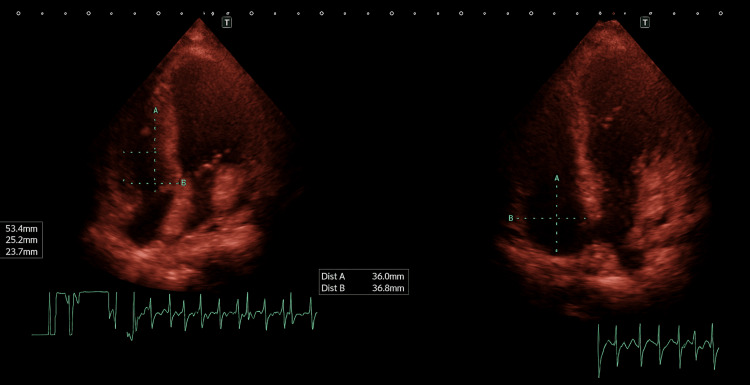
Transthoracic echocardiogram (TTE) Transthoracic echocardiogram (TTE), parasternal long-axis view, demonstrating preserved biventricular systolic function with no evidence of right ventricular dilatation, dysfunction, or pulmonary hypertension. Findings were consistent with hemodynamic stability and absence of right ventricular strain at the time of evaluation.

Despite appropriate antibiotic therapy, hypoxemia persisted without clinical or radiologic evidence of improvement. Echocardiography revealed preserved biventricular systolic function without right-ventricular (RV) dilation or pulmonary hypertension (Figure 3). NT-proBNP (167 pg/mL), hs-troponin T (5.07 pg/mL), and procalcitonin (0.06 ng/mL) remained within postpartum reference ranges. Repeat lactate values remained ≤1 mmol/L.

**Figure 4:**
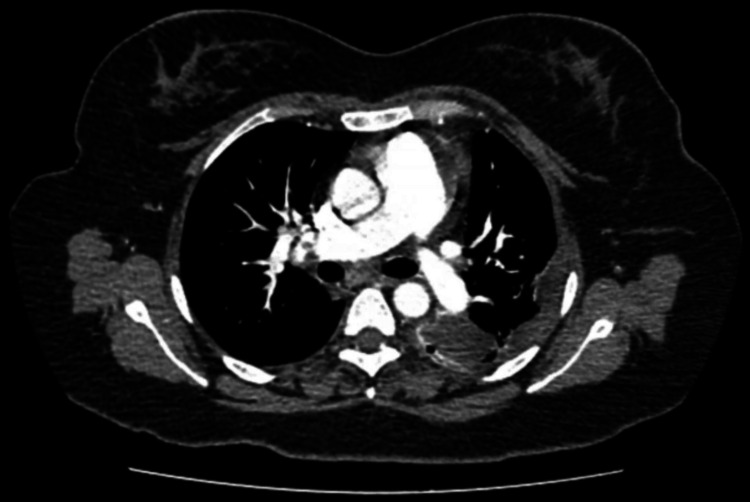
Contrast-enhanced computed tomography pulmonary angiography (CTPA) Contrast-enhanced computed tomography pulmonary angiography (CTPA) demonstrating extensive thrombus within the right main pulmonary artery and its lobar branches, as well as additional thrombi in the left pulmonary artery, consistent with massive pulmonary embolism.

Because her hypoxemia was disproportionate to the presumed infectious process and no alternative diagnosis explained her deterioration, CT pulmonary angiography (CTPA) was obtained. It demonstrated a large thrombus in the right main pulmonary artery extending into lobar branches and additional thrombi in the left pulmonary artery. These findings were consistent with massive pulmonary embolism by radiographic criteria (Figure 4). D-dimer testing was positive, but quantitative values were unavailable due to reagent limitations. Table 1 summarizes the evolution of hematologic and inflammatory markers.

**Table 1:** Evolution of hematological, inflammatory, and coagulation parameters during hospitalization Abbreviations: Hb, hemoglobin; WBC, white blood cell count; CRP, C-reactive protein; INR, international normalized ratio; NT-proBNP, N-terminal pro–B-type natriuretic peptide; hs-Troponin T, high-sensitivity cardiac troponin T ↑ Elevated value relative to the reference range. CRP was not measured on day 6; procalcitonin remained within the normal range (0.06 ng/mL, reference <0.5 ng/mL).

Hospital day	Hb (g/dL)	WBC (×10³/µL)	Platelets (×10³/µL)	CRP (mg/dL)	Procalcitonin (ng/mL)	D-dimer	INR	NT-proBNP (pg/mL)	hs-Troponin T (pg/mL)
Day 1 (ER)	13.8	19.0	611	2.05 ↑	–	Positive	1.0	–	–
Day 3	10.0	15.7	504	–	–	–	–	–	–
Day 5	–	–	–	–	–	–	–	167	5.07
Day 6	10.7	12.4	560	–	0.06	–	1.08	–	–
Day 9	10.5	11.8	570	–	–	–	3.59	–	–
Day 12	10.8	10.5	530	–	–	–	–	–	–

Intervention

Her Pulmonary Embolism Severity Index (PESI) was 92 (Class III). Three Hestia criteria were positive, consistent with intermediate-high-risk PE. Systemic thrombolysis was avoided due to the elevated postpartum bleeding risk, and catheter-directed therapy (CDT) was selected through multidisciplinary discussion.

Pulmonary angiography confirmed a large thrombotic burden with a mean pulmonary artery pressure of 50 mmHg. Mechanical aspiration thrombectomy was performed, followed by infusion of 50 mg alteplase over two hours.

**Figure 5:**
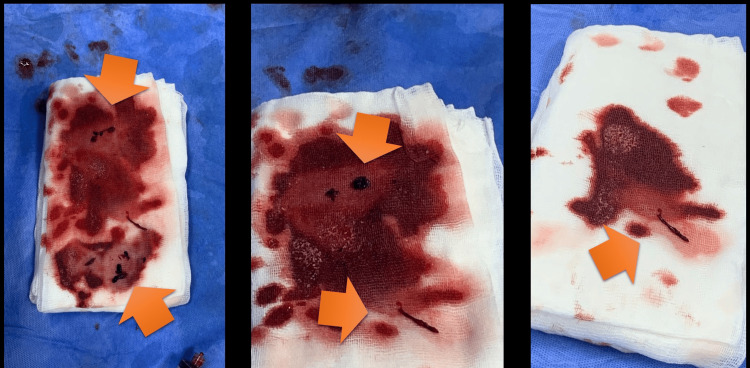
Extracted pulmonary thrombi following catheter-directed thrombectomy Gross appearance of thrombotic material aspirated from the right pulmonary artery during the procedure, showing fresh, red, and friable clots consistent with acute pulmonary embolism.

**Figure 6:**
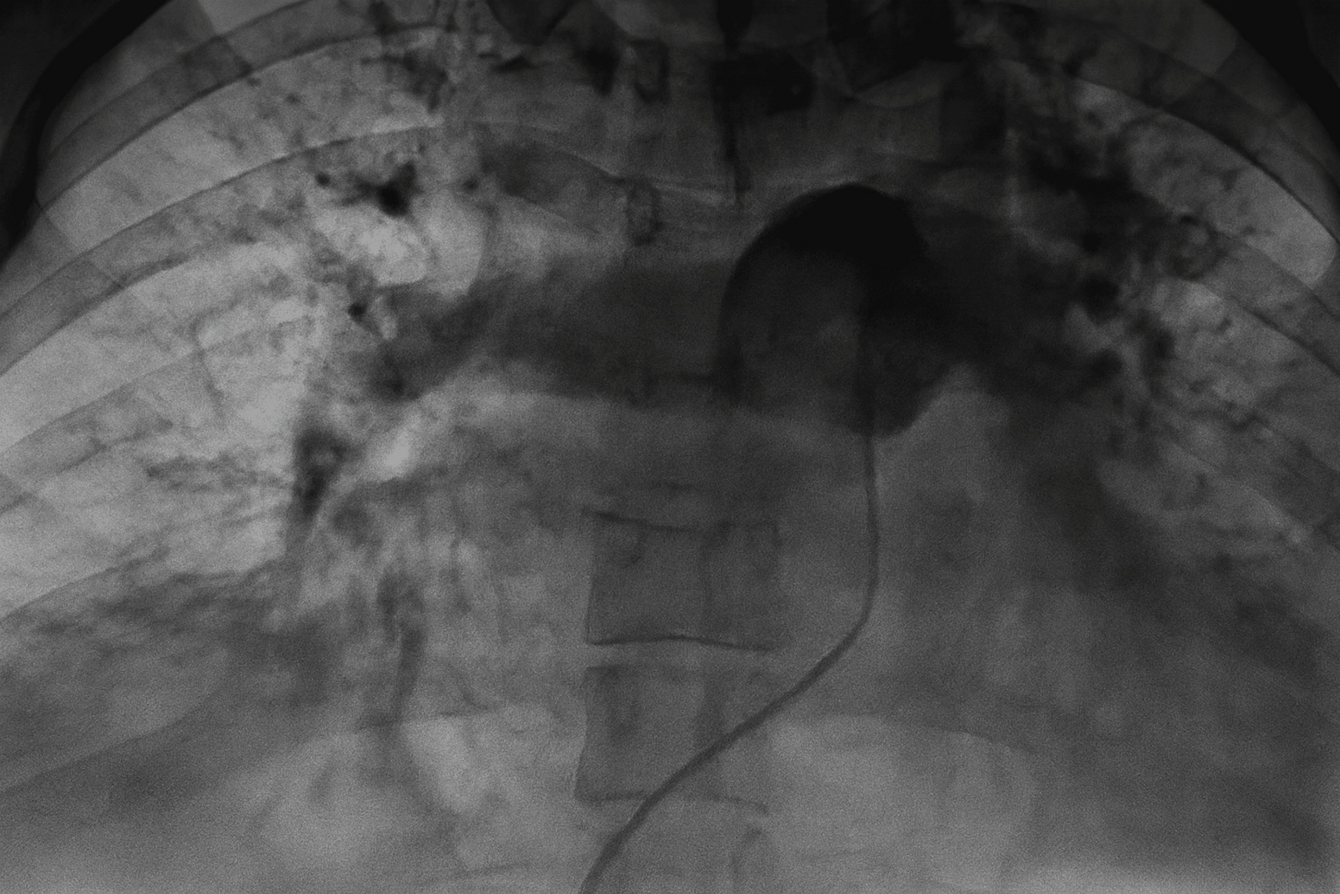
Post-thrombectomy pulmonary angiography Post-thrombectomy pulmonary angiography demonstrating complete opacification of the right pulmonary artery, with restoration of flow through its lobar and segmental branches. These findings confirm successful reperfusion and a reduction in mean pulmonary artery pressure from 50 mmHg to 35 mmHg compared with pre-procedure measurements.

Figure 5 shows the extracted thrombotic material. Figure 6 and Video 1 show the post-thrombectomy angiographic findings. Post-procedural angiography demonstrated complete reperfusion with a decrease in mean pulmonary artery pressure to 35 mmHg. No bleeding or procedural complications occurred.

**Video 1:** Post-thrombectomy pulmonary angiography Angiographic sequence demonstrating restored perfusion of the right pulmonary artery after catheter-directed aspiration thrombectomy and low-dose alteplase infusion.

Post-procedural course

The patient experienced rapid improvement in oxygenation and respiratory effort and remained hemodynamically stable without vasopressor support. Anticoagulation was transitioned from low-molecular-weight heparin to warfarin with therapeutic INR targets per APS guidelines. Hydroxychloroquine was initiated due to a strong clinical suspicion of antiphospholipid syndrome (APS) and its emerging role in thrombosis prevention. Follow-up echocardiography showed normal RV size and function.

**Figure 7:**
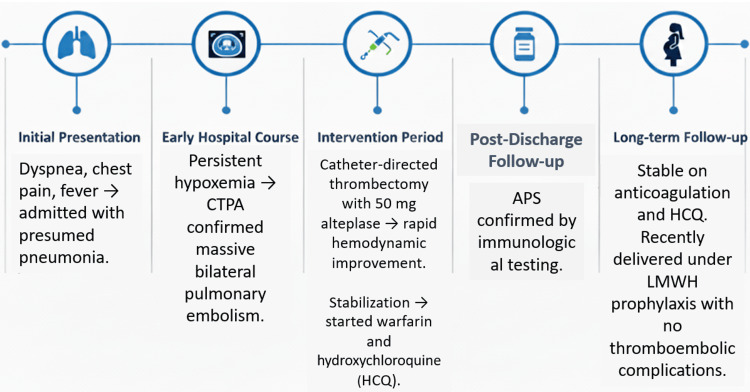
Clinical timeline Clinical timeline summarizing the patient’s course from symptom onset to diagnosis, intervention, and postpartum follow-up Abbreviations: CTPA, computed tomography pulmonary angiography; APS, antiphospholipid syndrome; LMWH, low-molecular-weight heparin; HCQ, hydroxychloroquine

Repeat antibody testing performed ≥ 12 weeks later confirmed persistent anticardiolipin and anti-β2 glycoprotein I antibody positivity, establishing APS. Over nearly three years of follow-up, she remained stable on warfarin and hydroxychloroquine. She later conceived while on LMWH prophylaxis and completed the pregnancy without thromboembolic or obstetric complications. Figure 7 summarizes her clinical trajectory.

## Discussion

This case illustrates the diagnostic and therapeutic challenges of managing massive postpartum PE, particularly when APS is the underlying etiology. The postpartum period carries the highest risk for venous thromboembolism, with estimates showing a 20-60-fold increase compared with non-pregnant women [4,8,9]. The patient initially presented with pleuritic chest pain, dyspnea, and leukocytosis, symptoms that closely mimic pneumonia, leading to diagnostic anchoring and a delay in obtaining CTPA. This highlights a key clinical lesson: postpartum patients with unexplained hypoxemia require early reconsideration of thromboembolic disease, even when infectious findings appear plausible.

Timely recognition of postpartum PE also requires adherence to updated diagnostic pathways such as those outlined in the 2019 ESC guidelines [7].

A critical learning point is that early use of standardized PE rule-out tools, such as the Wells criteria, would have classified the patient as “likely,” thereby prompting immediate CTPA and potentially preventing diagnostic delay [10]. This reinforces the vulnerability of postpartum patients to misdiagnosis when respiratory symptoms and radiographic abnormalities mimic infection.

Caution is warranted when interpreting D-dimer levels in the postpartum period, as physiologic hypercoagulability frequently produces elevated values and reduces diagnostic specificity. In this case, the D-dimer test was reported as positive, but a quantitative value was unavailable due to reagent limitations. Nonetheless, in the context of persistent hypoxemia, normal procalcitonin, and lack of improvement despite antibiotics, a positive D-dimer should lower the threshold for suspecting PE and favor expedited imaging.

Echocardiography was normal despite an extensive thrombotic burden on subsequent CTPA, illustrating a known limitation: right-ventricular (RV) dilation and dysfunction may be absent early or in patients with preserved cardiopulmonary reserve. Hemodynamic data obtained during pulmonary angiography, including a markedly elevated mean pulmonary artery pressure of 50 mmHg, confirmed severe pulmonary hypertension, emphasizing the importance of integrating invasive and noninvasive parameters in risk stratification.

CDT was selected to mitigate the increased postpartum hemorrhagic risk associated with systemic thrombolysis. Evidence from SEATTLE II and other series supports CDT as an effective reperfusion strategy with lower bleeding risk relative to systemic thrombolysis, while enabling rapid hemodynamic improvement [5,6]. Consistent with published outcomes, this patient experienced complete angiographic reperfusion with a reduction in pulmonary artery pressure to 35 mmHg, without bleeding or procedural complications.

APS provided a unifying explanation for the patient's severe placental disease (preeclampsia, IUGR, neonatal death) and the subsequent thromboembolic event. Diagnosis was confirmed by persistent anticardiolipin and anti-β2GPI antibody positivity on repeat testing at ≥12 weeks. Long-term management included warfarin and hydroxychloroquine. Hydroxychloroquine may reduce thrombotic events in APS by modulating platelet activation, endothelial function, and complement pathways [3]. This approach aligns with current EULAR recommendations for APS management [11]. The patient’s subsequent successful pregnancy under LMWH prophylaxis further underscores the importance of individualized, multidisciplinary long-term management.

This case highlights the need for standardized postpartum PE diagnostic pathways incorporating risk-stratification tools, early CTPA for persistent or unexplained hypoxemia, and coordinated multidisciplinary care. Early thrombophilia evaluation should be considered in women with severe placental dysfunction, recurrent pregnancy loss, or early-onset hypertensive disorders.

As a single-patient experience, generalizability is limited. However, this case adds meaningful clinical insight into diagnostic pitfalls in postpartum PE, supports the role of CDT in high-risk postpartum patients, and reinforces the need for further research and protocol development, particularly in the context of APS-associated PE.

## Conclusions

Massive PE in the postpartum period is a rare but potentially life-threatening event that requires a high index of suspicion, particularly in patients with obstetric comorbidities such as preeclampsia or fetal growth restriction. This case underscores the importance of early risk assessment and consideration of underlying thrombophilias, including APS, when clinical improvement is limited or findings are atypical.

CDT represents a valuable therapeutic alternative when systemic thrombolysis is contraindicated due to high postpartum hemorrhage risk. In this patient, CDT resulted in rapid hemodynamic improvement without bleeding complications, supporting its potential role in selected high-risk postpartum cases.

A coordinated multidisciplinary approach is essential for both acute management and long-term follow-up, including individualized anticoagulation strategies and pregnancy planning. Although findings from a single case cannot be generalized, this report contributes to the growing evidence supporting CDT in postpartum PE and highlights the need for standardized protocols and further research, particularly in patients with APS overlap.
